# The importance of pyramidal tract integrity for cortical plasticity and related functionality in patients with multiple sclerosis

**DOI:** 10.3389/fneur.2023.1266225

**Published:** 2023-11-24

**Authors:** Carolin Balloff, Philipp Albrecht, Arved-Sebastian Stucke, Lina Scala, Sveva Novello, Christian Johannes Hartmann, Sven Günther Meuth, Alfons Schnitzler, Iris-Katharina Penner, Stefan Jun Groiss

**Affiliations:** ^1^Department of Neurology, Medical Faculty and University Hospital Düsseldorf, Heinrich Heine University, Düsseldorf, Germany; ^2^Department of Neurology, Kliniken Maria Hilf GmbH, Mönchengladbach, Germany; ^3^Institute of Clinical Neuroscience and Medical Psychology, Medical Faculty and University Hospital Düsseldorf, Heinrich Heine University, Düsseldorf, Germany; ^4^Department of Neurology, Inselspital, Bern University Hospital, University of Bern, Bern, Switzerland; ^5^Neurocenter Düsseldorf, Düsseldorf, Germany

**Keywords:** cortical plasticity, motor function, repetitive transcranial magnetic stimulation, quadripulse stimulation, pyramidal tract integrity

## Abstract

**Background:**

Cortical plasticity induced by quadripulse stimulation (QPS) has been shown to correlate with cognitive functions in patients with relapsing-remitting multiple sclerosis (RRMS) and to not be reduced compared to healthy controls (HCs).

**Objective:**

This study aimed to compare the degree of QPS-induced plasticity between different subtypes of multiple sclerosis (MS) and HCs and to investigate the association of the degree of plasticity with motor and cognitive functions. We expected lower levels of plasticity in patients with progressive MS (PMS) but not RRMS compared to HCs. Furthermore, we expected to find positive correlations with cognitive and motor performance in patients with MS.

**Methods:**

QPS-induced plasticity was compared between 34 patients with PMS, 30 patients with RRMS, and 30 HCs using linear mixed-effects models. The degree of QPS-induced cortical plasticity was correlated with various motor and cognitive outcomes.

**Results:**

There were no differences regarding the degree of QPS-induced cortical plasticity between HCs and patients with RRMS (*p* = 0.86) and PMS (*p* = 0.18). However, we only found correlations between the level of induced plasticity and both motor and cognitive functions in patients with intact corticospinal tract integrity. Exploratory analysis revealed significantly reduced QPS-induced plasticity in patients with damage compared to intact corticospinal tract integrity (*p* < 0.001).

**Conclusion:**

Our study supports the notion of pyramidal tract integrity being of more relevance for QPS-induced cortical plasticity in MS and related functional significance than the type of disease.

## 1 Introduction

Neuroplasticity represents an important mechanism of the human brain to overcome disease-induced changes and impairment in the communication of neuronal networks. It does not only facilitate learning and memory (1) but also environmental adaptation and thus reflects an indispensable prerequisite for recovery and rebuilding of neuronal connections after brain injury and brain disease (2).

In multiple sclerosis (MS), a decline in motor and cognitive performance is the consequence of increased structural damage, finally leading to a network collapse impeding the brain's capability to reorganize (3). Thus, interventions able to promote brain plasticity to regain and/or preserve functions are of tremendous clinical and scientific interest and need. However, potential therapeutic interventions can only be investigated using reliable biomarkers of plasticity with high functional relevance, one of which may be repetitive transcranial magnetic stimulation (rTMS). Depending on the applied frequency, rTMS can change neural excitability by inducing effects similar to long-term potentiation (LTP) and long-term depression (LTD) (4). Many rTMS protocols exist and a protocol called quadripulse stimulation (QPS) (5) is supposed to promote LTP in healthy subjects with the lowest variability (6–8).

Recently, we were able to show that cortical plasticity can be induced by QPS of the motor cortex in patients with relapsing-remitting MS (RRMS) (9). In this cohort, plasticity induced by our QPS protocol was significantly associated with information processing speed, visuospatial learning and short-term memory, and with clinical disability. Correspondingly, cortical plasticity was higher in subjects with preserved cognitive function than in those presenting cognitive deficits. Compared to healthy controls (HCs), our overall mildly affected group of RRMS patients presented with similar levels of cortical plasticity (9).

Even though these findings indicate that QPS-induced plasticity could reflect global synaptic plasticity beyond the motor cortex, research is actually limited to patients with RRMS, neuropsychological performance, and clinical disability. Thus, to extend our knowledge and understanding in terms of clinical relevance and prognostic value of the QPS method, it is required to study its potential in different disease types and its relevance for motor functions as well.

In the present study, we, therefore, analyzed the correlation between the degree of synaptic plasticity with motor functions of the upper and lower extremities as well as with cognitive outcomes for processing speed and visuospatial short-term memory and learning in different types of MS and matched HCs. Furthermore, we compared the degree of QPS-induced plasticity between HCs and different MS subtypes. Based on the previously described association of cortical plasticity and clinical disability in patients with RRMS (9), we hypothesized QPS-induced plasticity to positively correlate with motor outcomes across all disease types. Based on the results from our first RRMS cohort, we further expected to find positive correlations with cognitive performance in patients with progressive MS (PMS).

To the best of our knowledge, only one study has investigated LTP- or LTD-like plasticity induced by rTMS in patients with PMS so far (10). Plasticity was shown to be reduced in patients with primary progressive MS (PPMS) compared to stable patients with RRMS and HCs. Patients with PPMS neither showed LTP-like effects following intermittent theta burst stimulation (iTBS) nor LTD-like effects following continuous theta burst stimulation (cTBS) (10). It was argued that these findings may be due to excitotoxicity neuronal damage and loss of a sufficient LTP-like response in patients with PPMS (11).

Even though these findings indicate that reduced or even absent synaptic plasticity may be an important factor driving clinical deterioration in patients with PPMS, the sample size of the PPMS group was too small (*n* = 12) to generalize the findings, and both iTBS and cTBS typically show high rates of non- or even opposite responders as well as high intra- and inter-individual variability (12). Furthermore, no data on the degree of cortical plasticity in patients with secondary progressive MS (SPMS) have been published until now.

Despite limited comparability between different rTMS protocols, both the aforementioned protocols and QPS aim to induce either LTP or LTD. Thus, in line with the previous findings and the fact that patients with PMS typically express more disability than patients with RRMS (13, 14), we expected to find similar results using QPS as in the previous study (10). Specifically, we hypothesized plasticity to be reduced in patients with PMS but not in RRMS compared to HCs.

## 2 Materials and methods

The design and methods of the study have been described in detail elsewhere (9). In the following paragraphs, we therefore only summarize the most relevant information to understand the design of the study as well as any deviations from our previous publication.

### 2.1 Subjects

Data were collected between May 2018 and October 2022 at the Department of Neurology at the University Hospital in Düsseldorf, Germany. The inclusion criterion was a diagnosis of definite MS according to the revised McDonald criteria (15). The exclusion criteria were as follows: (1) history of diseases of the central or peripheral nervous system other than MS, (2) history of psychiatric diseases potentially affecting cognition other than remitted depressive episodes, (3) presence of any contraindication for transcranial magnetic stimulation (TMS), (4) history of drug or alcohol abuse, (5) age of < 18 years. Based on the same exclusion criteria, age-, sex-, and education-matched HCs were recruited from an internal database of interested HCs as well as friends and family members of faculty members of the University Hospital Düsseldorf. A TMS safety screening questionnaire (16) was carried out, and informed written consent was obtained by all persons before participation. The ethical committee of the medical faculty of the Heinrich Heine University Düsseldorf (study number 2018-16) reviewed and approved the study, which was carried out in accordance with the Declaration of Helsinki.

### 2.2 Experimental design and data assessment

Details of the experimental design have been described in our previous publication (9). To summarize, a short neuropsychological assessment, including the Rao-adapted version of the Symbol Digit Modalities Test (SDMT) (17, 18), the Brief Visuospatial Memory Test-Revised (BVMT-R) (19), and patient-reported outcome measures of fatigue (Fatigue Scale for Motor and Cognitive Functions) (20), depression, and anxiety (Hospital Anxiety and Depression Scale) (21), was administered. Furthermore, the nine-hole peg test (NHPT) was applied as a functional outcome of manual dexterity, and the timed 25-foot walk test (T25FW) served as a measure of ambulation (22). The Expanded Disability Status Scale (EDSS) was determined by an experienced neurologist as an indicator of overall disability (23).

Change in motor-evoked potential (MEP) amplitudes at the right first dorsal interosseous muscle following 30 min of QPS-5 stimulation (5) served as a measure of LTP-like synaptic plasticity. MEPs were evoked by single-pulse monophasic TMS and were adjusted to be ~0.5 mV before the QPS-5 intervention to ensure comparability across subjects. In total, 12 MEPs were averaged for analysis. The same stimulation intensity was used to record MEPs post-QPS intervention for a total of 60 min. An average of 12 MEPs was calculated. However, on average, one MEP per subject was excluded at each time of assessment due to voluntary muscle activity and/or artifacts.

To assess pyramidal tract integrity, MEP latency was measured by single-pulse TMS. Participants were told to maintain a contraction of ~30% of the maximum voluntary activity at the target muscle for 10 consecutive trials, while they were stimulated with an intensity of 140% of their individual active motor threshold. The mean latency of the ten trials was used for analyses (24).

### 2.3 Statistical analyses

Since there are no published data on QPS-induced plasticity in patients with PMS so far, the number of enrolled subjects was based on the number of eligible patients with PMS and matched patients with RRMS/HCs. The comparison of demographic and clinical characteristics was conducted using IBM SPSS Statistics (version 28). All other analyses were carried out in R studio (version 2022.12.0), and statistical tests were considered significant based on α < 0.05.

Clinical and demographic characteristics were compared between groups using the Kruskal–Wallis test for continuous variables because data were non-normally distributed in at least one group per variable. MS-specific continuous characteristics (e.g., EDSS) were compared between patients with RRMS and PMS using the Mann–Whitney *U-*test due to non-normal distribution. Fisher's exact test was used to compare categorical data between groups. Significant omnibus tests were followed by Dunn's test or pairwise Fisher's exact test to identify which specific group(s) differed from the others. We report significant pairwise group differences based on uncorrected and Bonferroni–Holm-corrected *p'*-values (25).

To improve standardization and ensure comparability across our studies, the maximum change in MEP amplitude after QPS was used as our measure of synaptic plasticity.

Due to the presence of outliers, associations between QPS-induced plasticity and functional readouts (BVMT-R, SDMT, NHPT, and T25FW) were investigated by Spearman's rank correlation coefficients of these measures with the difference between the maximum of the six mean post-MEPs and the pre-MEP amplitude (ΔMEP) separately in each group. Uncorrected one-tailed *p*-values and Bonferroni–Holm-corrected *p'*-values are reported (25, 26). Data inspection revealed no clear linear relationship between the NHPT, T25FW, and ΔMEP in either group. Therefore, we did not further analyze linear relationships for these parameters but used generalized additive models (GAMs) to explore more complex linear and non-linear relationships with the “mgcv” package in both patient groups. In addition, we explored the following types of splines: thin plate, penalized cubic, cyclic cubic, shrinkage cubic, and p. Models were compared against the regular linear model using the “anova” function.

Linear mixed-effects models were calculated using the “nlme” package to compare the degree of induced plasticity between HCs, patients with RRMS, and patients with PMS. In line with our previous study (9), the increase of MEP amplitude following QPS was analyzed by comparing the maximum of the six mean post-MEP amplitude against the mean MEP amplitude before QPS (~0.5 mV). A random slope for the intervention (pre/post-QPS) was added to the fixed effects of the intervention (pre/post-QPS), group (HCs, RRMS, and PMS), and their interaction. This accounted for both the dependency of pre- and post-MEP amplitudes within subjects due to repeated measurements as well as for the variability of the interventional effect.

The basic model predicting the MEP amplitude included the fixed effects of the intervention (pre/post-QPS) and group (HCs, RRMS, and PMS), as well as their interaction. To account for the subject-dependent variability in response to the intervention, a random slope for the intervention (pre/post-QPS) was included. For our research question, the interactions of post-QPS^*^group were most relevant since significant interactions would indicate a significant difference in the degree of plasticity between the corresponding groups. Specifically, we hypothesized a significantly reduced increase of MEP amplitude in patients with PMS compared to HCs and patients with RRMS. Due to this directed hypothesis, one-tailed confidence intervals and *p*-values were conducted for the factor post-QPS^*^PMS. All other confidence intervals and *p*-values were based on two-tailed analysis.

In line with our previous study (9), age, depression, anxiety, fatigue, and MEP latency, as well as their interactions were separately added to the model. Models including covariates were tested against the basic model described above based on likelihood-ratio tests. Models including covariates with missing data were compared based on the Akaike information criterion. The variance inflation factor (cutoff value of ≥5) was used to investigate collinearity. All models were estimated using the “restricted maximum likelihood method” because it results in a more precise estimation of standard errors in smaller samples (27). We only report the model with the best fit.

Lastly, we conducted exploratory analyses. In the first analysis, we divided the PMS group into SPMS and PPMS and repeated linear mixed-effect modeling with four instead of three groups to avoid systematic errors possibly evolving from the merger of both PMS groups. In the second and third analyses, we divided all patients with MS into two groups based on pyramidal tract integrity as measured by cortical latency. MEP latency is a measure of corticospinal conduction velocity and may be prolonged in patients with MS with pyramidal tract affection (28). Based on the clinical norms of the University Hospital Düsseldorf and to avoid misclassifications as “pathological,” MEP latencies of < 24.5 ms were considered normal. Spearman's rank correlation coefficients of ΔMEP with all functional readouts were calculated for both groups, following the same approach as described above. Uncorrected two-tailed *p*-values and Bonferroni–Holm-corrected (25, 26) *p'*-values are reported. Lastly, we compared the degree of induced plasticity between patients with pathological vs. normal cortical latency using linear mixed-effects models. The model computation followed the same procedures as described above. However, latency was not included as a covariate in the second analysis as patients were divided into two groups based on this variable.

## 3 Results

### 3.1 Demographic and clinical characteristics

**Figure 1:**
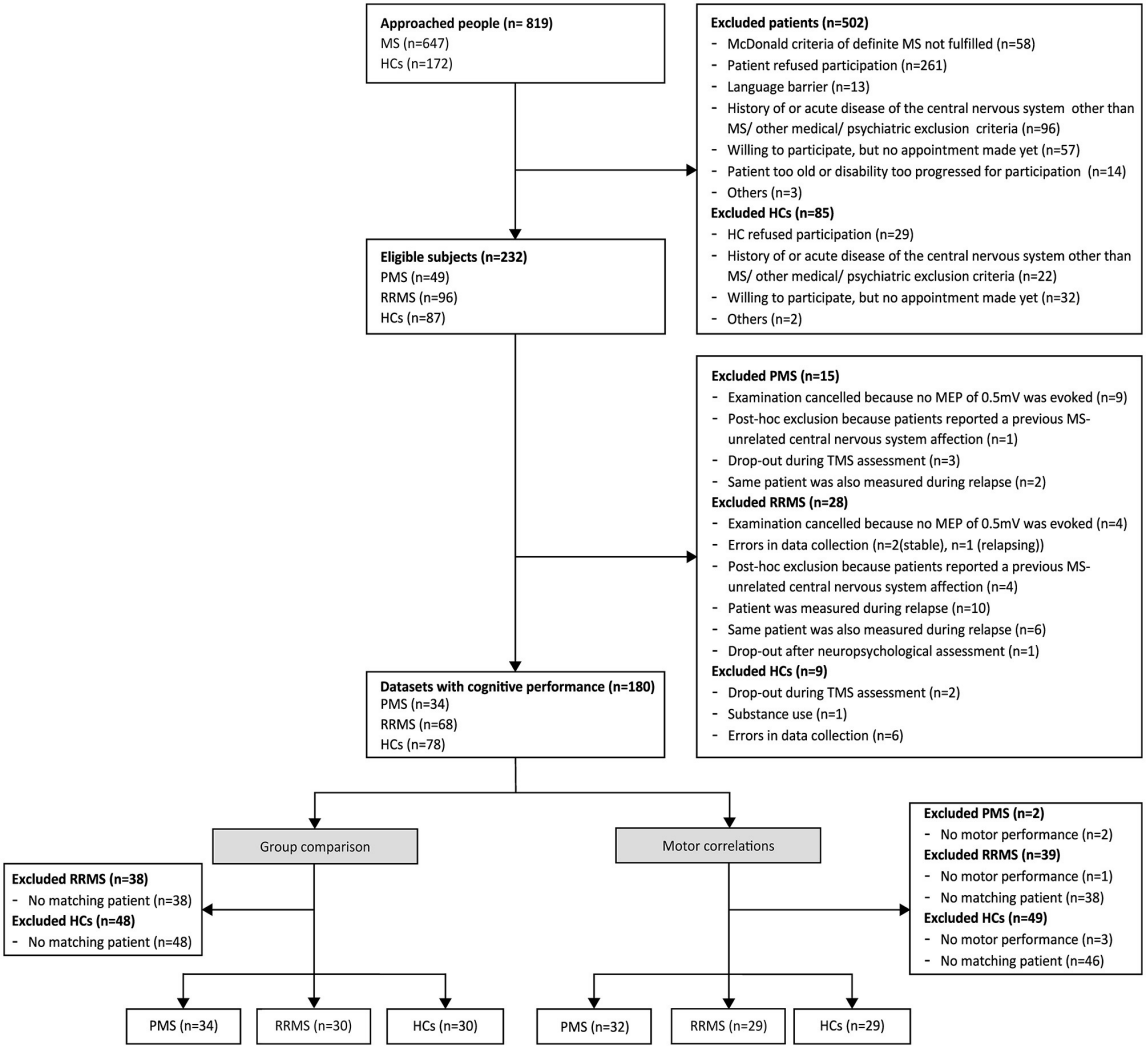
Flowchart of the enrollment of subjects. The flowchart presents the number of subjects at each step of the study. Since data on motor performance were missing in some subjects, slightly different subgroups were used to analyze the relationship between motor outcomes and cortical plasticity and to compare the degree of induced plasticity between disease types. HCs, healthy controls; MS, multiple sclerosis; MEP, motor-evoked potential; RRMS, relapsing-remitting multiple sclerosis; PMS, progressive multiple sclerosis (primary and secondary progressive multiple sclerosis); TMS, transcranial magnetic stimulation.

Out of 819 people approached, a total of 34 patients with PMS (14 PPMS, 20 SPMS), as well as 30 matched patients with RRMS and 30 matched HCs were included (Figure 1). Demographic characteristics of each subgroup are presented in Table 1 and were compared between groups. As expected, patients with PMS were significantly more disabled than patients with RRMS and HCs, indicated by higher EDSS, longer cortical latency, worse performance in SDMT, BVMT-R, NHPT, and T25FW, and higher rates of unemployment. Furthermore, patients with PMS had higher active and resting motor thresholds than HCs and required higher stimulation intensity to evoke an MEP amplitude of ~0.5 mV compared to both patients with RRMS and HCs. Patients with RRMS required higher stimulation intensity to evoke an MEP amplitude of ~0.5 mV compared to HCs and performed worse on the NHPT as well as T25FW. The distribution of motor and cognitive fatigue was comparable between both patient groups, but, as expected, more patients described clinical levels of fatigue than HCs. Due to missing data in the T25FW and NHPT, 32 patients with PMS, 29 matched patients with RRMS, and 29 HCs were included in further analyses of the relationship between motor performance and cortical plasticity.

**Table 1:** Sample characteristics.

**Characteristic**	**PMS (*n =* 34)**	**RRMS (*n =* 30)**	**HCs (*n =* 30)**	***p*-value**
Sex, *N* (%), female	16 (47)	16 (53)	16 (53)	0.90
Handedness, *N* (%), right^a^	32 (97)	27 (90)	27 (90)	0.55
Age, Md (IQR), years	52.5 (12)	48.5 (9)	53 (15)	0.25
Education, Md (IQR), years	15 (5)	16 (6)	17 (5)	0.21
Employment, *N* (%), yes	16 (47)	23 (77)	26 (87)	**0.002** ^**b**^
AMT, Md (IQR), % MSO	45.5 (13)	44.5 (12)	39 (5)	**0.005** ^**c**^
RMT, Md (IQR), % MSO	55.5 (15)	51.5 (20)	48 (7)	**0.007** ^**d**^
MEP 0.5 mV, Md (IQR), % MSO	81 (35)	66.5 (32)	58 (12)	**< 0.001** ^**e**^
MEP latency, Md (IQR), ms	25.4 (6.4)	23.4 (2.5)	22.8 (2.8)	**< 0.001** ^**f**^
ΔPost-Pre MEP amplitude, Md (IQR), mV	0.2 (0.5)	0.5 (0.6)	0.5 (0.7)	0.27
**BVMT-R**				
Total learning score, Md (IQR)	19 (13)	23.5 (13)	28 (6)	**< 0.001** ^**g**^
z-score, Md (IQR)	−1.2 (2.4)	−0.1 (2.5)	0.81 (1.2)	**< 0.001** ^**h**^
**SDMT**				
Correct items, Md (IQR)	42.5 (16)	51.5 (24)	54.5 (20)	**< 0.001** ^**i**^
z-score, Md (IQR)	−1.2 (1.5)	−0.1 (1.9)	0.43 (2.0)	**< 0.001** ^**i**^
**Nine-hole peg test**				
Time to complete, Md (IQR), seconds	25.3 (9.9)	22.1 (5.4)	18.9 (2.2)	**< 0.001** ^**j**^
**25-foot walk test**				
Time to complete, Md (IQR), seconds	6.4 (4.2)	4.5 (1.8)	3.5 (1.1)	**< 0.001** ^**k**^
**HADS**, ***N*** **(%), clinical**				
Anxiety	1 (3)	5 (17)	0 (0)	**0.03** ^**l**^
Depression	5 (15)	4 (13)	0 (0)	0.07
**FSMC**, ***N*** **(%), mild/moderate/severe**				
Motor	1 (3)/5 (15)/25 (74)	5 (17)/4 (13)/15 (50)	5 (17)/1 (3)/2 (7)	**< 0.001** ^**m**^
Cognitive	4 (12)/5 (15)/ 16 (47)	3 (10)/7 (23)/ 12 (40)	4 (13)/3 (10)/2 (7)	**< 0.001** ^**m**^
**MS specific characteristics**				
Disease duration, Md (IQR), years	12.2 (16)	13.5 (10)		0.86
EDSS, Md (IQR)^n^	5.0 (3.0)	2.0 (2.3)		**< 0.001**
DMT at time of assessment, *N* (%)				0.35
None	6 (18)	5 (17)		
Natalizumab	4 (12)	7 (23)		
Ocrelizumab	21 (62)	12 (40)		
Fingolimod	1 (3)	1 (3)		
Cladribine	1 (3)	2 (7)		
Alemtuzumab	1 (3)	0 (0)		
Glatiramer acetate	0 (0)	2 (7)		
Dimethyl fumarate	0 (0)	1 (3)		

*p*-values < 0.05 (two-sided) were considered significant and are in boldface. HCs, healthy controls; PMS, patients with progressive multiple sclerosis; RRMS, patients with relapsing-remitting multiple sclerosis; Md, median; IQR, interquartile range; AMT, active motor threshold; RMT, resting motor threshold; MEP, motor-evoked potential; MEP 0.5 mV, stimulation intensity producing a reliable MEP of ~0.5 mV; MSO, maximal stimulator output; BVMT-R, Brief Visuospatial Memory Test-Revised; SDMT, Symbol Digit Modalities Test; HADS, Hospital Anxiety and Depression Scale; FSMC, Fatigue Scale of Motor and Cognition; EDSS, Expanded Disability Status Scale; DMT, disease-modifying therapy. ^a^Missing data: *n* = 1 (PMS). ^b^Uncorrected and Bonferroni–Holm-corrected pairwise chi-square tests revealed significant differences between HCs and PMS as well as PMS and RRMS. ^c^Uncorrected Dunn's pairwise comparisons revealed significant differences between all groups. After the Bonferroni–Holm correction, only the difference between HCs and PMS remained significant. ^d^Uncorrected and Bonferroni–Holm corrected Dunn's pairwise comparisons revealed a significant difference between HC and PMS. ^e^Uncorrected and Bonferroni–Holm corrected Dunn's pairwise comparisons revealed significant differences between all groups. ^f^Uncorrected and Bonferroni–Holm corrected Dunn's pairwise comparisons revealed significant differences between HCs and PMS as well as PMS and RRMS. Missing data: *n* = 5 (PMS: *n* = 2, RRMS: *n* = 1, HCs: *n* = 2). ^g^Uncorrected and Bonferroni–Holm corrected Dunn's pairwise comparisons revealed significant differences between HCs and PMS as well as PMS and RRMS. ^h^Uncorrected Dunn's pairwise comparisons revealed significant differences between all groups. After the Bonferroni–Holm adjustment, only the difference between HC and PMS remained significant. Calculation of z-scores based on the norms provided in the BVMT-R manual (19). ^i^Uncorrected and Bonferroni–Holm corrected Dunn's pairwise comparisons revealed significant differences between HCs and PMS, as well as PMS and RRMS. Z-scores were calculated based on German norms (17). ^j^Uncorrected and Bonferroni–Holm corrected Dunn's pairwise comparisons revealed significant differences between all groups. Missing data: *n* = 5 (2 PMS and 3 HCs). ^k^Uncorrected and Bonferroni–Holm corrected Dunn's pairwise comparisons revealed significant differences between all groups. Patients, who were unable to walk the required distance (*n* = 8 [4 PMS, 4 RRMS]), were assigned the following time to complete: maximum time to complete (total MS Sample) + 1.645^*^standard deviation (total MS sample). Missing data: *n* = 5 (2 PMS, 3 HCs). ^l^Uncorrected and Bonferroni–Holm corrected pairwise chi-square tests revealed no significant differences between any groups. Classification as “clinical” based on scores ≥ 11 (21). ^m^Uncorrected and Bonferroni–Holm corrected pairwise chi-square tests revealed significant differences between HCs and PMS as well as HCs and RRMS. Classification (mild, moderate, and severe) based on cutoffs provided in the manual of the FSMC (20). ^n^Missing data: *n* = 1 (PMS). MS, multiple sclerosis.

### 3.2 Differences in QPS-induced plasticity between patients with PMS and matched patients with RRMS and HCs

**Figure 2:**
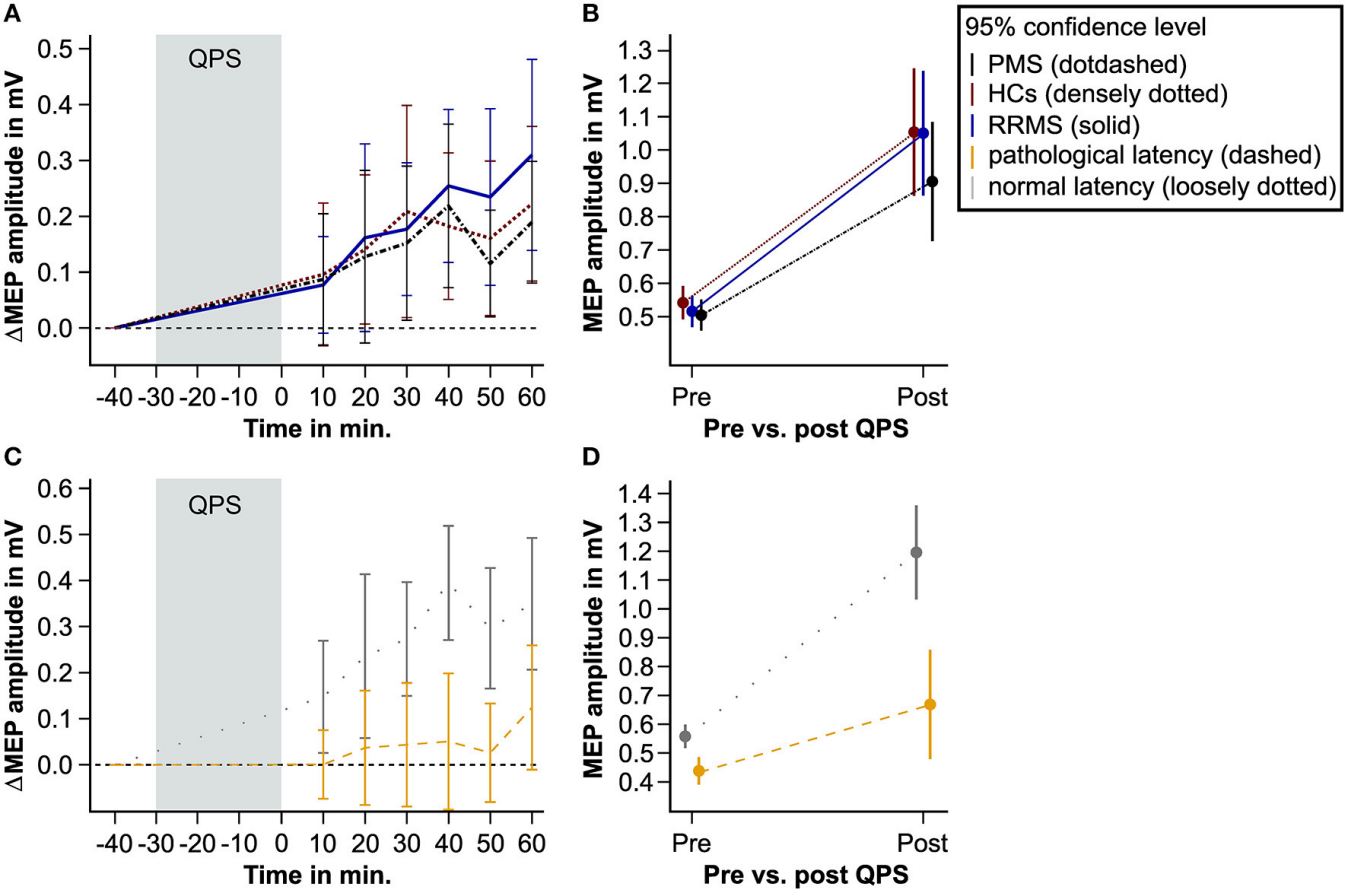
QPS-induced plasticity in patients with MS and matched HCs. This figure shows the level of QPS-induced plasticity in patients with PMS (black dot-dashed line), patients with RRMS (blue solid line), and HCs (red densely dotted line). Furthermore, it illustrates QPS-induced plasticity in patients with pathological (yellow dashed line) and normal MEP latency (gray loosely dotted line). **(A, C)** show the averaged difference between the pre- and post-QPS MEP amplitude in mV per time point and group. **(B, D)** illustrate the predicted MEP amplitude in mV based on the fixed effects of the linear mixed models pre- and post-QPS comparing HCs, PMS, and RRMS **(B)** and patients with pathological and normal MEP latency **(D)**. QPS, quadripulse stimulation; MS, multiple sclerosis; MEP, motor-evoked potential; HCs, healthy controls; RRMS, patients with relapsing-remitting multiple sclerosis; PMS, patients with progressive multiple sclerosis.

In all study groups, i.e., HCs, patients with RRMS, and patients with PMS, MEP amplitudes significantly increased after the QPS intervention. However, there was no difference in ΔMEP between groups (Table 2, Figures 2A, B). A significant main effect of cortical latency was revealed. Across all groups and both times of MEP measurement, longer latencies were associated with lower MEPs (Table 2).

**Table 2:** Multivariable linear mixed-effects model of MEP amplitude over time in HCs, patients with RRMS, and patients with PMS.

**Fixed effects**					**Random effects**
	β**-coefficient (95% CI)**	**SE**_b_	***t-*** **value**	***p***	**SD**
Intercept	+0.54(+0.49; +0.59)^a^	0.03	+21.28	**< 0.0001**	
Pre-QPS	Reference				
Post-QPS	+0.51 (+0.33; +0.69)^a^	0.09	+5.73	**< 0.0001**	
HCs	Reference				
RRMS	−0.03 (−0.09; +0.04)	0.03	−0.75	0.45	
PMS	−0.04 (−0.11; +0.04)	0.04	−1.02	0.31	
Age	−0.01 (−0.04; +0.02)	0.01	−0.77	0.45	
Latency	−0.04 (−0.07; −0.01)^a^	0.02	−2.45	**0.02**	
Post-QPS^*^RRMS	+0.02 (−0.23; +0.27)	0.18	+0.18	0.86	
Post-QPS^*^PMS	−0.11 (–∞; +0.09)	0.12	−0.91	0.18	
Age^*^Latency	+0.03 (−0.00; +0.06)	0.02	+1.92	0.06	
Subject^*^Pre-QPS					0.10
Subject^*^Post-QPS					0.51
Residual					0.08

Two-tailed 95% confidence intervals and *p*-values are displayed for all factors except for the primary variable of interest (post-QPS^*^PMS). For this factor, testing our hypothesis, that synaptic plasticity is reduced in patients with PMS, one-tailed 95% confidence intervals and *p*-values are reported. *p*-values of < 0.05 are in boldface. *t*- and *p*-values are based on the asymptotic Wald test. Continuous variables (age, latency) centered at sample mean. R^2^(conditional) = 0.97. R^2^(marginal) = 0.32. Adjusted intraclass correlation coefficient = 0.95. QPS, quadripulse stimulation; HCs, healthy controls; RRMS, patients with relapsing-remitting multiple sclerosis; PMS, patients with progressive multiple sclerosis. ^a^Indicates statistical significance. SE_b_, standard error of β-coefficient.

To ensure that our statistical analyses were not fraught with systematic errors by merging PPMS and SPMS into one group of PMS, additional analyses were conducted separating patients with PPMS from patients with SPMS. The results did not reveal any significant differences in the degree of cortical plasticity (Supplementary Table 1, Supplementary Figure 1).

### 3.3 Association between functional readouts and QPS-induced plasticity

**Figure 3:**
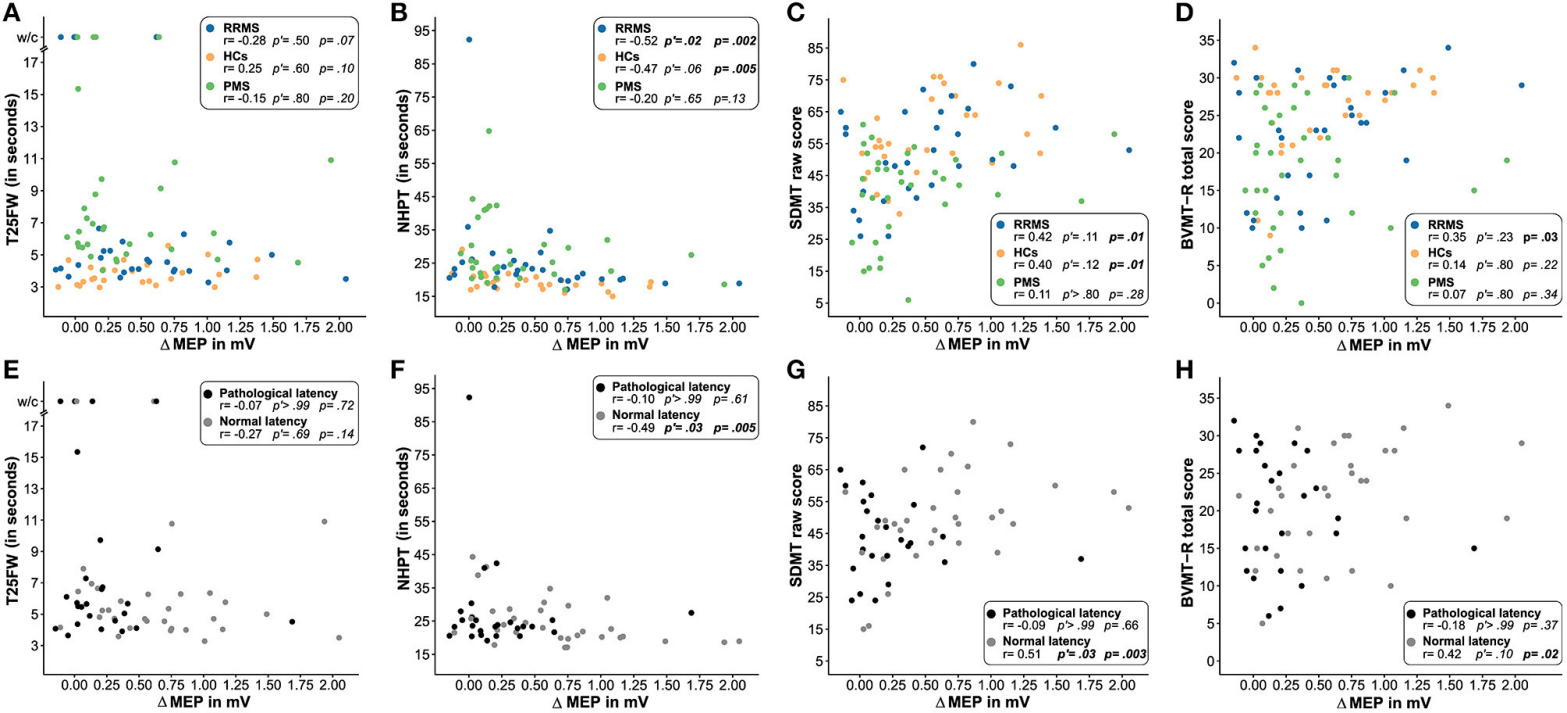
Association of the degree of induced plasticity with functional readouts. This figure illustrates the association of the degree of induced plasticity (ΔMEP) with the T25FW **(A, E)**, NHPT **(B, F)**, SDMT **(C, G)**, and BVMT-R **(D, H)** in patients with RRMS (blue), PMS (green), and HCs (orange) and in patients with pathological (gray) and normal (black) cortical latency. Uncorrected one-tailed *p*-values and Bonferroni–Holm-corrected *p'*-values are reported for **(A–D)**. For **(E–H)**, uncorrected two-tailed *p*-values and Bonferroni–Holm-corrected *p'*-values are reported. *P*-values of < 0.05 are displayed in bold face. HCs, healthy controls; RRMS, patients with relapsing-remitting multiple sclerosis; PMS, patients with progressive multiple sclerosis; T25FW, timed 25-foot walk test; w/c, wheelchair; NHPT, nine-hole peg test; BVMT-R, Brief Visuospatial Memory Test-Revised; SDMT, Symbol Digit Modalities Test; MEP, motor-evoked potential; ΔMEP, difference between the maximum of the six mean MEP amplitude after repetitive quadripulse stimulation and the MEP amplitude before stimulation.

Concerning motor functions as measured by the T25FW and NHPT, ΔMEP correlated significantly with the time to complete the NHPT in patients with RRMS and HCs but not in patients with PMS. When controlling for multiple testing, significance was lost in HCs. No correlation was found between ΔMEP and the T25FW in either of the three groups. Despite the statistically significant correlation coefficients for the NHPT, data inspection revealed no clear linear relationship in either group for both measures of motor function (Figures 3A, B). We, therefore, modeled non-linear associations between both motor functions and ΔMEP, which did, however, not improve the model fit in either group.

Concerning cognitive functions, ΔMEP correlated significantly with the SDMT raw score in patients with RRMS and HCs but not in patients with PMS. Controlling for multiple testing, the association did not reach statistical significance in any group. ΔMEP also significantly correlated with the BVMT-R total score in patients with RRMS but missed statistical significance when controlling for multiple testing. No correlation was found for the BVMT-R in patients with PMS and HCs (Figures 3C, D).

Splitting the two patient groups by pyramidal tract integrity as measured by cortical latency, those patients with normal MEP latency showed significant correlations of ΔMEP with the NHPT, SDMT raw score, and the BVMT-R total score but not with the T25FW. The association remained statistically significant after the Bonferroni–Holm correction for the NHPT and SDMT raw score. Patients with pathological MEP latency did not show any correlation between ΔMEP and motor and cognitive readouts (Figures 3E–H).

Linear mixed-effects modeling revealed that QPS-induced plasticity was significantly reduced in patients with pathological compared to patients with normal cortical latency (Figures 2C, D, Table 3).

**Table 3:** Multivariable linear mixed-effects model of MEP amplitude over time in patients with normal vs. patients with pathological cortical latency.

**Fixed effects**					**Random effects**
	β**-coefficient (95% CI)**	**SE**_b_	***t*** **-value**	**p**	**SD**
Intercept	+0.56 (+0.52; +0.60)^a^	0.02	+26.82	**< 0.0001**	
Pre-QPS	Reference				
Post-QPS	+0.64 (+0.49; +0.79)^a^	0.08	+8.36	**< 0.0001**	
Normal cortical latency	Reference				
Pathological cortical latency	−0.12 (−0.18; −0.06)^a^	0.03	−3.75	**< 0.001**	
Post-QPS^*^Pathological cortical latency	−0.41 (−0.64; −0.17)^a^	0.12	−3.49	**< 0.001**	
Subject^*^Pre-QPS					0.09
Subject^*^Post-QPS					0.49
Residual					0.08

Two-tailed 95% confidence intervals, and *p*-values are displayed. *p*-values of < 0.05 are in boldface. *t*- and *p*-values are based on the asymptotic Wald test. R^2^(conditional) = 0.97. R^2^(marginal) = 0.42. Adjusted intraclass correlation coefficient = 0.95. QPS, quadripulse stimulation. ^a^Indicates statistical significance. SE_b_, standard error of β-coefficient.

## 4 Discussion

This is the first study comparing QPS-induced plasticity of the motor cortex between HCs and different types of MS. Our study has two main findings. First, QPS-induced cortical plasticity did not differ between HCs and matched patients with RRMS and PMS. Second, we revealed intact corticospinal tract integrity as a prerequisite for the correlation between the degree of cortical plasticity and both motor and cognitive functions.

We found relevant associations between QPS-induced cortical plasticity and both motor and cognitive functions in patients with MS. However, this association was limited to cases in which MEP latencies, representing corticospinal conduction velocity (28), were normal. Importantly, exploratory analysis revealed that significantly higher degrees of plasticity were induced in these patients compared to patients with prolonged MEP latency. The relevance of structural integrity of the pyramidal tract for rTMS-induced cortical plasticity and learning abilities has already been shown in neurologically healthy subjects, suggesting rTMS to be valuable in identifying patients at risk of developing dementia (29). This is in line with our current results revealing pyramidal tract integrity as a requirement for the correlation of cortical plasticity and motor and cognitive function. Axonal cortical neurodegeneration with pyramidal tract affection may lead to lower synaptic density or activity and may therefore be relevant for plasticity impairment and functional deterioration in MS.

Importantly, the SDMT was conducted verbally, ensuring that no motor functions, apart from speech, were involved. Inaccuracies in the drawings of the BVMT-R due to motor dysfunctions (e.g., wriggly lines) were not considered in the scoring of the test. Therefore, motor dysfunction is unlikely to be responsible for the results. Nonetheless, we have conducted *post hoc* analyses comparing SDMT and BVMT-R results between patients with and without prolonged MEP latency. As expected, no significant differences were revealed between the groups. Thus, we conclude that pyramidal tract integrity could be an important factor to be controlled for in future plasticity studies in MS, e.g., by separately analyzing rTMS-induced plasticity in patients with normal and pathological cortical latency or even introducing pathological MEP latencies as an exclusion criterion.

Interestingly, only one of our motor outcomes, namely, the NHPT but not the T25FW, correlated with the degree of cortical plasticity. NHPT measures represented motor function from the left hemisphere to the right hand for which QPS-induced cortical plasticity of the left hemisphere corresponded to, while the results of the T25FW could have been influenced by other networks such as the cerebellar system and/or lesions in the spinal cord. In addition to age, performance in the T25FW has recently been associated with normalized deep gray matter volume, whereas the NHPT has been associated with normalized gray matter volume and cognitive performance (30). Thus, NHPT, BVMT-R, and SDMT may require more similar networks than T25FW, BVMT-R, and SDMT. In line with this, NHPT, BVMT-R, and SDMT were associated with cortical plasticity but not the T25FW.

We did not find cortical plasticity to be reduced in patients with PMS. This result contradicts the assumption that the progressive phase of the disease is characterized by insufficient compensatory reserve to balance out the negative consequences of inflammation and neurodegeneration (31). Furthermore, it is in contrast to an earlier study comparing TMS-induced cortical plasticity using iTBS and cTBS between patients with RRMS and PPMS (10). Patients with RRMS showed preserved plasticity, while it was absent in patients with PPMS after iTBS, which is supposed to induce LTP-like plasticity. Interestingly, cortical plasticity still turned out to be altered in patients with RRMS since cTBS, which originally had been supposed to induce LTD-like plasticity, led to a reversal of plasticity and induced LTP-like effects (10). The authors suggested that platelet-derived growth factor (PDGF) may play a substantial role in LTP induction in patients with MS. Although we did not measure PDGF levels in the cerebrospinal fluid in our study, other reasons may account for the different results between these two studies. In the earlier study, MEP latency, RMT, and AMT were also significantly higher in patients with PPMS, suggesting relevant pyramidal tract affection in this group. Therefore, given the importance of pyramidal tract integrity revealed in the present study, the difference in induced cortical plasticity revealed in the earlier study (10) may have been driven by pyramidal tract affection rather than the type of MS. Furthermore, higher rates of variability have been described for iTBS and cTBS and verified also in direct comparison to QPS recently (6, 8, 12). Considering the low sample size of patients with PPMS in the previous study (*n* = 12) (10), alterations of induced plasticity in this group may have been an unsystematic result of high variability of previously used protocols rather than a systematic difference between disease types. Moreover, although recent TMS work postulated that loss of inhibition may be particularly important in SPMS (32), it has been shown that excitatory glutamatergic circuits may play a key role in MS pathology (33–35). In contrast to iTBS and cTBS, which influence both excitatory and inhibitory networks, QPS is supposed to selectively modulate excitatory glutamatergic cortical networks (5). Thus, the QPS protocol may induce LTP more efficiently in patients with MS and therefore may have yielded different results than the iTBS and cTBS protocols. Future studies should compare the effects of different rTMS protocols in patients with MS intraindividually to reveal the strengths and limitations of each protocol and thus increase the quality of future investigations of plasticity in MS.

Recruitment of patients with PMS for rTMS research may have some pitfalls, the most difficult being the relatively low prevalence rate compared to RRMS. Moreover, patients with PMS are typically more severely impaired (13, 14), impeding participation in studies with extensive protocols due to exhaustion or mobility issues. Despite these challenges, we included a sample that was almost three times as big (*n* = 34 vs. *n* = 12) as in the previous study by Mori et al. (10). We decided to summarize patients with PPMS and SPMS to one group of PMS to increase the statistical power since the clinical disease and pathophysiology appear to be similar (36). However, lower levels of white matter lesions and inflammation have been described for PPMS (31, 36), and it is still an open debate whether this disease subtype represents the same or a distinct disease entity. Therefore, we conducted an exploratory analysis, in which we compared QPS-induced plasticity in patients with PPMS and SPMS separately to matched HCs. However, we did not find any differences across groups, supporting the legitimacy of our approach to summarize the two progressive disease types.

Our study is not without limitations. Due to the cross-sectional design, no conclusions regarding the clinical relevance for disease progression can be drawn. Furthermore, the lack of imaging data prevents us from analyzing the impact of (sub)cortical lesions. Thus, we cannot rule out that MEP latencies may have not only been prolonged due to damaged pyramidal tracts but also due to abnormalities in the motor cortex. In addition, MEP latency may have been influenced by the participant's height and age. Patients with MS received different disease-modifying therapies and symptomatic medications, which potentially have impacted cortical excitability. Even though no systematic evaluation of the effects of different treatments on cortical excitability exists to the best of our knowledge, stabilizing effects of disease-modifying drugs on cortical excitability over time have been suggested in patients with PMS (37). Due to high variations in the stimulation protocols, target muscles, and study populations, different numbers of averaged trials to achieve reliable MEP assessments have been recommended (38–41). We chose to average 12 MEPs at each time of assessment to maintain a concise protocol and minimize participant fatigue. However, this number of average trials is at the lower end of the recommendations, and we might have improved the reliability of our findings by increasing the number of averaged MEPs. Lastly, baseline MEP amplitude was controlled to be ~0.5 mV in all patients. However, this amplitude could have been distributed at varying places on the recruitment curve for the different subjects (42), potentially causing ceiling effects in patients with impaired corticospinal tract integrity (43). Furthermore, QPS may have affected MEP size differentially depending on the stimulation intensity relative to the recruitment curve (5, 44).

Although overall cortical plasticity between RRMS and PMS was comparable on the group level, i.e., the degree of QPS-induced plasticity did not differ between them, it is plausible that there were disparities in the proportion of patients with corticospinal dysfunction and the extent of such dysfunction between groups. This is supported by the fact that PMS patients had longer MEP latencies compared to RRMS patients. In accordance with this, we identified associations between QPS-induced plasticity and behavioral outcomes only among patients with normal MEP latency, primarily those with RRMS, but not among patients with prolonged MEP latency, primarily those with PMS. In patients with prolonged MEP latency, it is conceivable that damage to the corticospinal tract exerted a more pronounced influence on QPS-induced plasticity, potentially overshadowing other associations, such as those between QPS-induced plasticity and behavioral measures. However, due to the exploratory character of this discovery in our study, we can only speculate about its neuropathological underpinnings, which warrant further investigation.

Despite these limitations, our study supports the notion of pyramidal tract integrity being of more relevance for QPS-induced cortical plasticity in MS and related functional significance than the amount of progression.

## Data availability statement

The raw data supporting the conclusions of this article will be made available by the authors, without undue reservation.

## Ethics statement

The studies involving humans were approved by ethical committee of the medical faculty of the Heinrich Heine University Düsseldorf. The studies were conducted in accordance with the local legislation and institutional requirements. The participants provided their written informed consent to participate in this study.

## Author contributions

CB: Conceptualization, Data curation, Formal analysis, Investigation, Methodology, Project administration, Visualization, Writing – original draft. PA: Conceptualization, Funding acquisition, Investigation, Methodology, Writing – original draft. A-SS: Data curation, Formal analysis, Investigation, Writing – review & editing. LS: Investigation, Writing – review & editing. SN: Investigation, Writing – review & editing. CH: Investigation, Writing – review & editing. SM: Resources, Writing – review & editing. AS: Resources, Writing – review & editing. I-KP: Conceptualization, Funding acquisition, Methodology, Writing – original draft. SG: Conceptualization, Funding acquisition, Investigation, Methodology, Writing – original draft.
