# Supplementary material for: The importance of pyramidal tract integrity for cortical plasticity and related functionality in patients with multiple sclerosis
Source: Front Neurol. 2023 Nov 24;14:1266225. doi: 10.3389/fneur.2023.1266225 (PMC10704601; doi:10.3389/fneur.2023.1266225)
Supplement: Supplementary file 1 [file Data_Sheet_1.pdf]

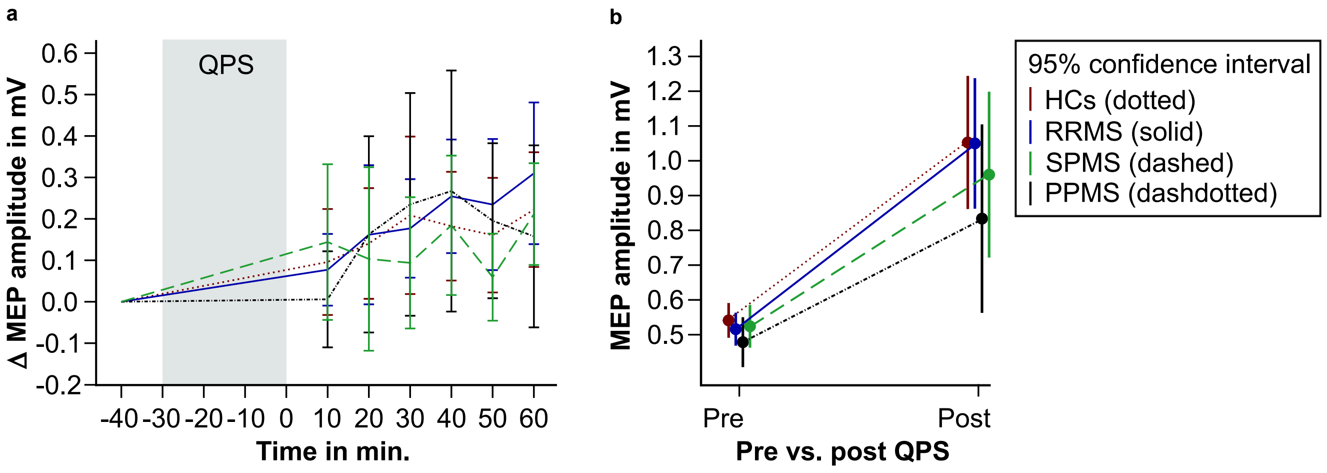

Supplementary Figure 1. QPS-induced plasticity separately for patients with PPMS and SPMS compared to matched pwRRMS and HCs.

This figure shows the level of QPS-induced plasticity in patients with PPMS (black dashdotted line), SPMS (green dashed line), RRMS (blue solid line), and HCs (red dotted line). Part A shows the averaged difference between the pre and post QPS MEP amplitude per time point in all groups. Part B illustrates the predicted MEP amplitude in mV based on the fixed effects of the linear mixed models pre and post QPS. QPS=Quadripulse stimulation; MEP=Motor evoked potential; HCs=Healthy Controls; RRMS=Relapsing-remitting multiple sclerosis; SPMS=Secondary progressive multiple sclerosis; PPMS= Primary progressive multiple sclerosis
