# Supplementary material for: The importance of pyramidal tract integrity for cortical plasticity and related functionality in patients with multiple sclerosis
Source: Front Neurol. 2023 Nov 24;14:1266225. doi: 10.3389/fneur.2023.1266225 (PMC10704601; doi:10.3389/fneur.2023.1266225)
Supplement: Supplementary file 2 [file Table_1.docx]

Supplementary Material

# Supplementary Table 1. Multivariable linear mixed-effects model of MEP amplitude over time in HCs, patients with RRMS, PPMS and SPMS

| **Fixed Effects** | | | | | **Random Effects** | |
| --- | --- | --- | --- | --- | --- | --- |
|  | *β-coefficient (95% CI)* | *SE_b_* | *t-value* | *p* | | *SD* |
| Intercept | +0.54 (0.49; 0.59)^a^ | 0.03 | +21.21 | **<.0001** | |  |
| Pre QPS | Reference |  |  |  | |  |
| Post QPS | +0.51 (+0.33; +0.69)^a^ | 0.09 | +5.71 | **<.0001** | |  |
| HCs | Reference |  |  |  | |  |
| RRMS patients | -0.03 (-0.09; +0.04) | 0.03 | -0.73 | .47 | |  |
| PPMS patients | -0.06 (-0.15; +0.03) | 0.05 | -1.38 | .17 | |  |
| SPMS patients | -0.02 (-0.10; +0.07) | 0.04 | -0.40 | .69 | |  |
| Age | -0.01 (-0.04; +0.02) | 0.01 | -0.54 | .59 | |  |
| Latency | -0.04 (-0.07; -0.01)^a^ | 0.02 | -2.46 | **.02** | |  |
| Post QPS*RRMS | +0.02 (-0.23; +0.27) | 0.13 | +0.18 | .86 | |  |
| Post QPS*PPMS | -0.16 (-0.47; +0.15) | 0.16 | -1.01 | .32 | |  |
| Post QPS*SPMS | -0.08 (-0.36; -0.08) | 0.14 | -0.53 | .60 | |  |
| Age*Latency | +0.03 (+0.00; +0.07)^a^ | 0.02 | +2.08 | **.04** | |  |
| Subject*Pre QPS |  |  |  |  | | 0.10 |
| Subject*Post QPS |  |  |  |  | | 0.51 |
| Residual |  |  |  |  | | 0.08 |

*Note.* Two-tailed 95% confidence intervals and *p*-values are displayed. *p*-values <.05 are in boldface. *t*- and *p*-values are based on asymptotic Wald test. Continuous variables (age, latency) centered at sample mean. R²(conditional)=0.97. R²(marginal)=0.32. Adjusted Intraclass Correlation Coefficient=0.95.

QPS= Quadripulse stimulation. HCs=Healthy Controls. RRMS= Relapsing remitting Multiple Sclerosis. PPMS= Primary progressive Multiple Sclerosis; SPMS= Secondary progressive Multiple Sclerosis. SE_b=_ Standard error of the β-coefficient.

^a^ indicates statistical significance.
